# Supplementary material for: Integrated analysis of the aqueous humor microbiome and lens capsule transcriptome in high myopia cataract: a pilot study
Source: Front Med (Lausanne). 2026 Jun 16;13:1845205. doi: 10.3389/fmed.2026.1845205 (PMC13314463; doi:10.3389/fmed.2026.1845205)
Supplement: Supplementary file 3 [file Data_Sheet_1.zip › supplemental_material/about_GTDB.pdf]

GTDB 数据库介绍

1. GTDB 数据库是什么？

GTDB 是由澳大利亚昆士兰大学化学与分子生物科学学院生态基因组学中心的团队开发，**基于公认的平均核苷酸一致性（ANI）标准来设置物种界限的基因组分类数据库。该数据库提供了从界到种完整的分类学信息，解决了细菌和古菌基因组的分类问题。**初版发表在 Nature Biotechnology（IF 68.164），更新后再次发表在 Nature Biotechnology、Nature Microbiology(IF 30.964) 等高水平期刊。

2. 与 RefSeq 什么关系？

GTDB 数据库中基因组直接来源于 NCBI，版本也与 RefSeq 库相对应，例如 GTDB 最新版为 Release 08-RS214，其中 RS214 表示对应 RefSeq 214 版本。**在 RefSeq 基础之上进行了基因组质量控制并提供了更合理的分类信息。**

3. 最新版数据库包含多少物种和基因组？

当前版本共包含 40 多万（402,709）个基因组，涉及 8.5 万（85,205）个物种，详细统计结果如下：

细菌和古菌各分类水平数的统计（不是基因组数量统计）

	细菌	古菌	总计
门	161	20	181
纲	488	60	548
目	1,624	148	1,772
科	4,262	508	4,772
属	19,153	1,586	20,739
种	80,789	4,416	85,205

4. GTDB 基因组都有哪些来源？

GTDB 中的基因组都可以从 NCBI 数据库中查询下载到。**基因组主要有纯培养组装的基因组、宏基因组测序组装的基因组和单细胞测序组装的基因组构成**。下图是相关统计（分离纯培养（Exclusively isolates）、宏基因组或单细胞（Exclusively MAGs and/or SAGs）、分离纯培养或宏基因组或单细胞（Isolate and environmental genomes））：

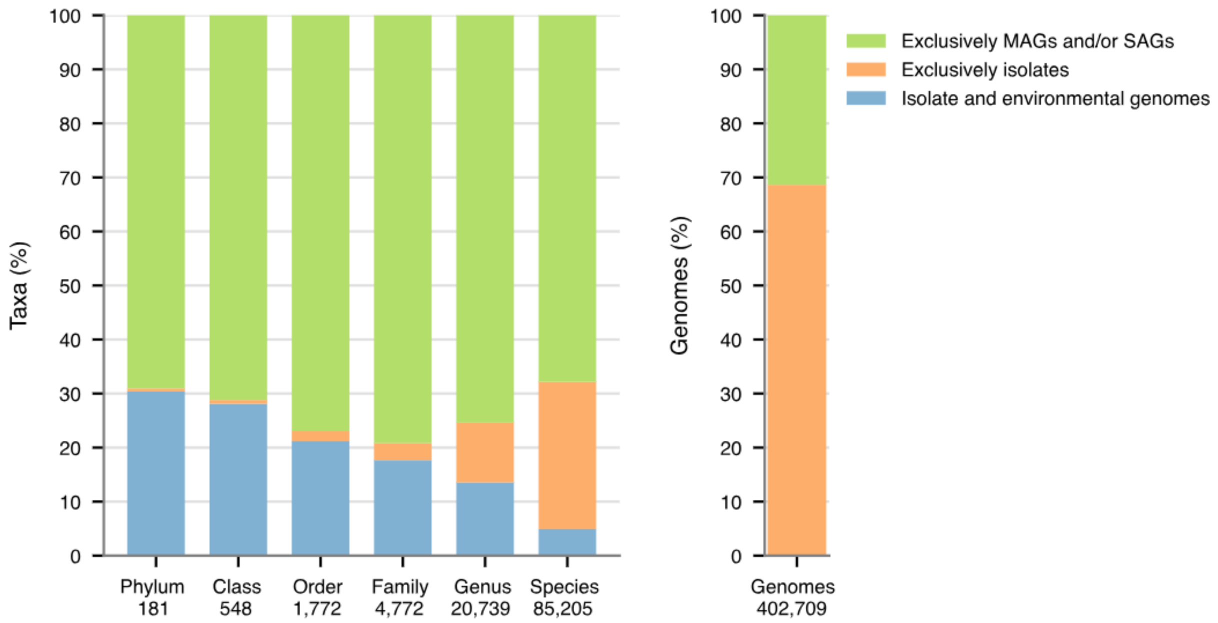

各分类水平下基因组来源统计图

5. GTDB 中基因组的质量如何？

<u>GTDB 对基因组质量（组装完整性和污染比例）进行了严格控制</u>：使用 CheckM 进行污染控制，并基于 MIMAG 对基因组的完整性进行控制，入选标准为 完整性 - 5 * 污染比例 > 50，**<u>此外，为了保证数据库全面性，有部分低质量但属于重要物种的基因组，也一并纳入数据库中</u>**，详细情况见下图：

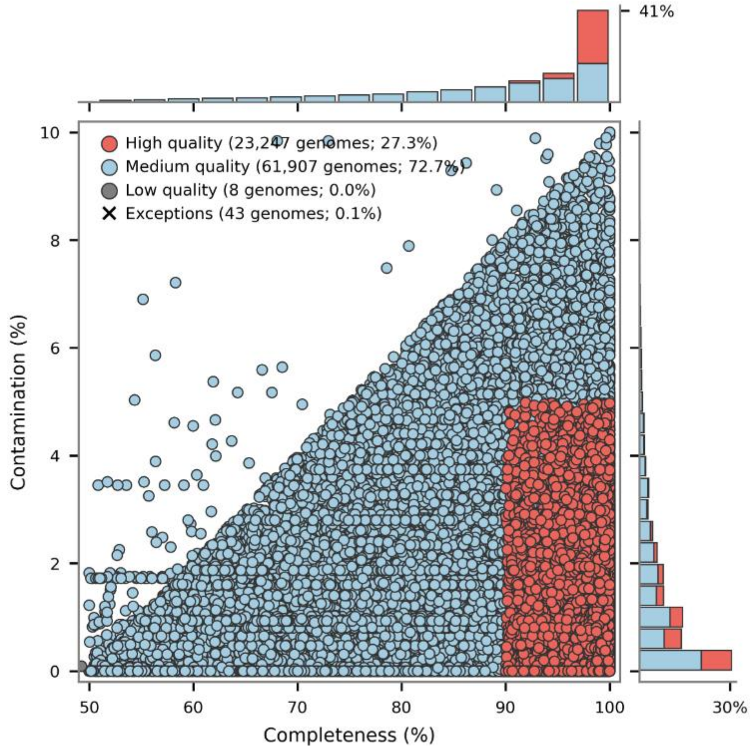

GTDB 数据库中基因组质量分布统计图

6. GTDB 和 NCBI 分类系统中有多少差异？

GTDB 对每一个基因组给出了一个分类信息，其中分为了三种情况：1. GTDB 与 NCBI 分类不同，标记为 Active change；2. NCBI 中无分类信息，标记为 Passive change；3. GTDB 与 NCBI 分类相同，标记为 Unchanged。详细统计如下图，可见 **GTDB 对 RefSeq 中大量未分类的 level 提供了合理的分类信息**：

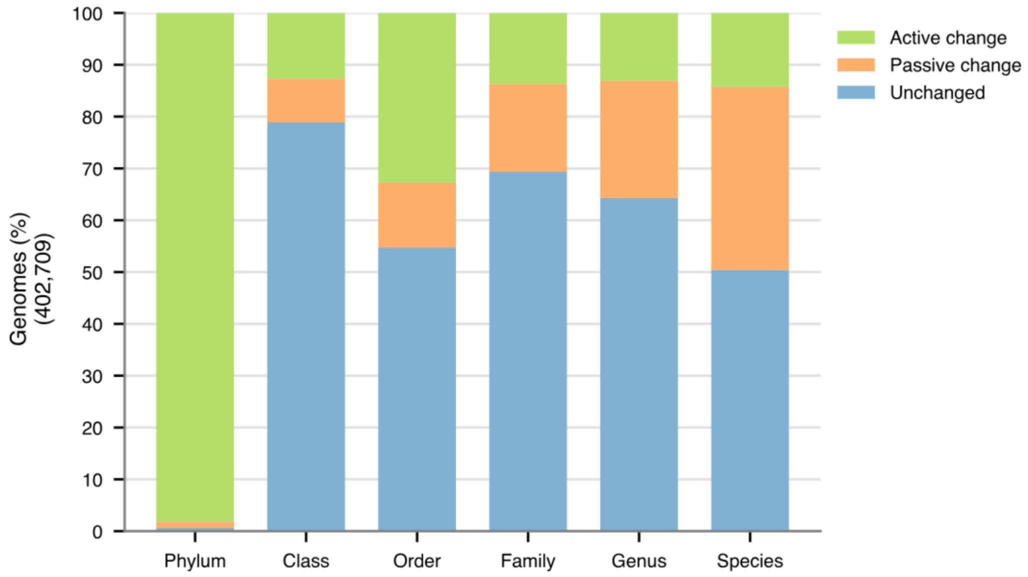

GTDB 与 NCBI（RefSeq）分类一致性统计图

7. GTDB 和 NCBI 分类系统中的微生物名如何对应？

由于两个分类系统的分类依据不同，物种名的对应关系也相对比较复杂，下面是两个分类系统中物种名的对应关系示意图，总结起来有四种：1. 一对一；2. 一对多；3. 多对一；4. 多对多。

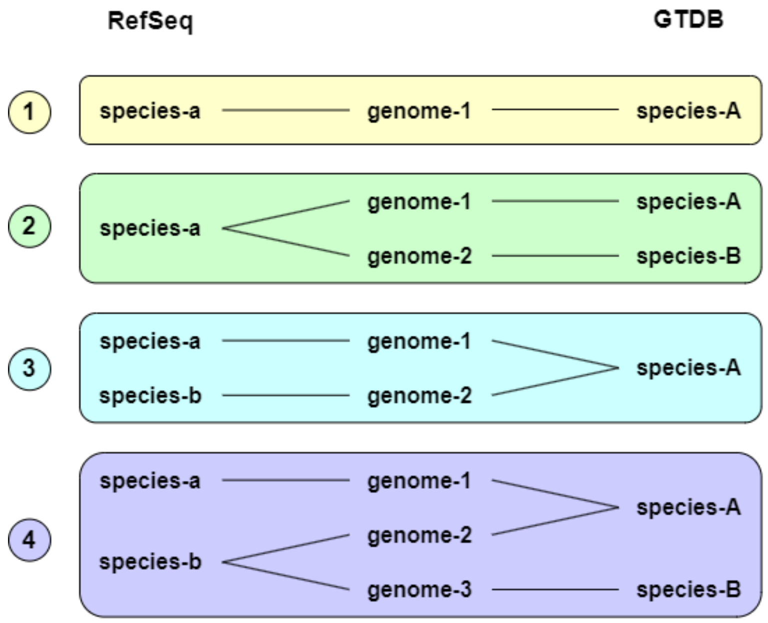

虽然对应关系复杂，但 GTDB 官网（https://gtdb.ecogenomic.org/）提供了与 NCBI 分类系统的对应关系查询入口，可以**直接在官网右上角的搜索框中输入 GCF/GCA 登录号或 NCBI 分类名或 GTDB 分类名进行查询**。

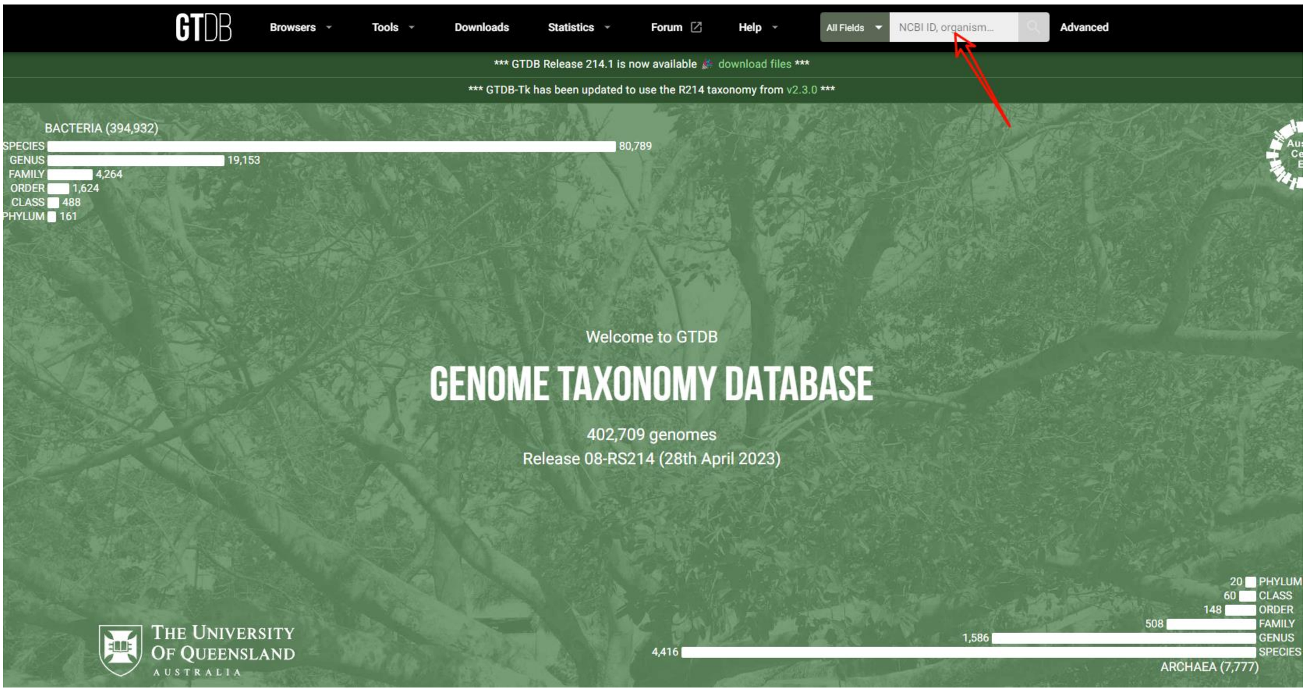

8. 2bRAD-M 产品为什么不选择 16S 分类系统/宏基因组分类系统？

现有 16S 和宏基因组分析流程/软件所用数据库，基本都是基于 NCBI 的分类系统构建的，而从上面的介绍中可以总结出 GTDB 数据库相比 NCBI（RefSeq）的四大特点：（1）基因组直接来源于 NCBI 的 RefSeq 库；（2）对基因组进行了严格的质量控制；（3）更合理、准确的分类方法；（4）填补了大量 NCBI 中未分类的 level。

2bRAD-M 基于种间差异 tag 进行物种鉴定，可处理痕量样本，对物种分类的准确度要求自然也更高，所以 2bRAD-M 产品默认选择 GTDB 数据库进行分析。此外，越来越多的 16S 和宏基因组分析软件开始兼容并使用 GTDB 数据库，例如 Qiime2、Metaphlan4 等。使用 GTDB 数据库将是微生物物种分类的大趋势。
